# Comparative Analysis of the Gut Microbiota of Three Sympatric Terrestrial Wild Bird Species Overwintering in Farmland Habitats

**DOI:** 10.3389/fmicb.2022.905668

**Published:** 2022-07-19

**Authors:** Zhiyuan Lu, Sisi Li, Min Wang, Can Wang, Derong Meng, Jingze Liu

**Affiliations:** ^1^Hebei Key Laboratory of Animal Physiology, Biochemistry and Molecular Biology, College of Life Sciences, Hebei Normal University, Shijiazhuang, China; ^2^College of Life Sciences, Cangzhou Normal University, Cangzhou, China

**Keywords:** Great Bustard, Common Crane, Common Coot, sympatric species, gut microbiota

## Abstract

The gut microbiota of wild birds are affected by complex factors, and cross-species transmission may pose challenges for the host to maintain stable gut symbionts. Farmland habitats are environments strongly manipulated by humans, and the environmental characteristics within a large area are highly consistent. These features provide the ideal natural conditions for conducting cross-species comparative studies on gut microbiota among wild birds. This study aimed to investigate and compare the gut microbiota of three common farmland-dependent bird species, Great Bustard (*Otis tarda dybowskii*), Common Crane (*Grus grus*), and Common Coot (*Fulica atra*), in a homogeneous habitat during the wintering period. The results indicated that under the combined action of similar influencing factors, the gut microbiota of different host species did not undergo adaptive convergence, maintained relatively independent structures, and exhibited host-driven signals. In addition, we also detected various pathogenic genera that may cause outbreaks of periodic infections among sympatric migratory birds. We conclude that phylosymbiosis may occur between some wild birds and their gut microbiota. Usage of non-invasive methods to monitor the changes in the gut microbiota of wild bird fecal samples has important implications for the conservation of endangered species.

## Introduction

The gut microbiota of wild birds are potentially shaped by diverse factors, such as genetics, environment, diet, and behavior (Pearce et al., 2017; Grond et al., 2018; Gillingham et al., 2019; Laviad-Shitrit et al., 2019). Unlike in birds, the microbial communities in most non-flying mammals are strongly correlated with host phylogeny (Song et al., 2020). The brood-parasitism model system clearly demonstrates that genetic components determine the gut microbiota of host and parasitic birds, suggesting that gut morphology and physiology might be important factors in generating interspecific differences (Lee et al., 2020). Comparative studies on the gut microbiota of multiple wild Neotropical birds have revealed that gut modifications and individual dietary differences shape the structure and variation of the gut microbiome, leading to the lack of phylogenetic symbiosis (Bodawatta et al., 2021). In addition, changes in habitat environment can significantly affect the composition of the gut microbiota of wild birds (Phillips et al., 2018; San Juan et al., 2020). American White Ibises along urban gradients exhibit a positive correlation between urban land cover and susceptibility to enteric pathogens (Murray et al., 2020). Moreover, the negative effects of urbanization have caused dysbiosis in the gut microbiota of sparrows (Teyssier et al., 2018). These species living in environments with severe anthropogenic disturbances face higher health risks than other species.

During the long-term development of agricultural practices, numerous wild bird species have adapted to, and now even depend on, farmland habitats, but the significance of farmland biodiversity is often overlooked (Li et al., 2020). During the winter, the green plants and ectothermic animals at mid or high latitudes hibernate or die under the stress of the low temperature, and the birds from various ecological niches gather to forage in farmlands (English et al., 2017; Li et al., 2021). Shared habitats may facilitate the interspecific spread of gut bacteria. It has been suggested that the gut microbiota of different species of migratory passerines become similar at the same stopover sites, and the changes become more pronounced with a longer stay (Lewis et al., 2017). The Hooded Crane and Bean Goose have overlapping niches during the wintering period and exhibit cross-species transmission of their gut bacteria (Yang and Zhou, 2021). Unlike resident bird species, migratory birds with complex travel patterns typically have a high diversity of gut microbes from various geographical regions and are also reservoirs for many pathogenic bacteria (Grond et al., 2014; Fu et al., 2020). Cross-species transmission hinders the host from maintaining stable gut symbionts and poses disease risks (Ryu et al., 2014). Therefore, comparative analysis of the gut microbiota, with special attention to the pathogenic composition, of overwintering farmland-dependent birds that share the same region may facilitate the risk monitoring for the conservation of endangered species.

In this study, we used three phylogenetically distance sympatric farmland wintering birds, aiming to investigate whether the composition of the gut microbiota is primarily host-driven or determined by exogenous factors. We focused on the Great Bustard, Common Coot, and Common Crane which share the same habitat in winter. The Great Bustard (*Otis tarda*) is a typical agricultural steppe bird that mainly feeds on green plants, grain seeds, and arthropods (Liu et al., 2018; Faragó, 2019). This species is the heaviest bird capable of flight, weighing up to 18 kg (Dunning 2008). It has been listed as a globally vulnerable species and China’s national Class I key protected animal. The eastern population of the Great Bustard (*Otis tarda dybowskii*) breed in Mongolia, south-east Russia, and north-east China (Collar et al., 2017), overwinter in the North China Plain, Guanzhong Plain, and Northeast Plain of China (Lu et al., 2021b), and migrate up to 2000 km, almost twice the recorded migratory distance of the nominate subspecies (Kessler et al., 2013). Only 1,200–2,200 Asian Great Bustards remain (Alonso, 2015), and there are gaps in the protection of the main wintering sites, which are highly susceptible to anthropogenic disturbance and face a high survival pressure (Alonso and Palacin, 2010; Mi et al., 2016, 2017). The Common Coot (*Fulica atra*) is a globally distributed waterfowl, widely dispersed and abundant in lakes and wetlands, and usually forages on plants and small animals in the water or along the shore (Cramp and Simmons, 1980; Perrow et al., 1997; Randler, 2006). It is partly sedentary and partly migratory (Harrison, 1982). The Common Crane (*Grus grus*) is a widespread wetland bird (Harris et al., 2000). It primarily breeds in mires and wetlands in forest-dominated areas (Leito et al., 2003), and its overwintering habitats are mainly located in stubble fields or agricultural lands (Li et al., 2021). It is a long-distance migratory bird (Ojaste et al., 2020), with a distribution range covering the entire northern Eurasia (Harris and Mirande, 2013).

During wintering, the Great Bustard, Common Coot, and Common Crane share the same habitat and feed mainly on plant seeds from farmlands and wastelands (Johnsgard, 1983; Mouronval et al., 2007; Liu et al., 2018). These species all have a complex digestive system with a developed cecum (Fanke et al., 2011; Crompton and Nesheim, 2016; Li et al., 2017) and share similar behavioral patterns, such as terrestrial living and group-living (Alonso et al., 2004; Mouronval et al., 2007; Lu et al., 2021a). Simplification of confounding factors can facilitate comparative analysis of the gut microbiota of wild bird species living under natural conditions (Lee et al., 2020), and a shared overwintering habitat provides an ideal condition to compare the adaptive changes in the gut microbiota of different hosts.

## Materials and Methods

### Fecal-Sample Collection

**Figure 1:**
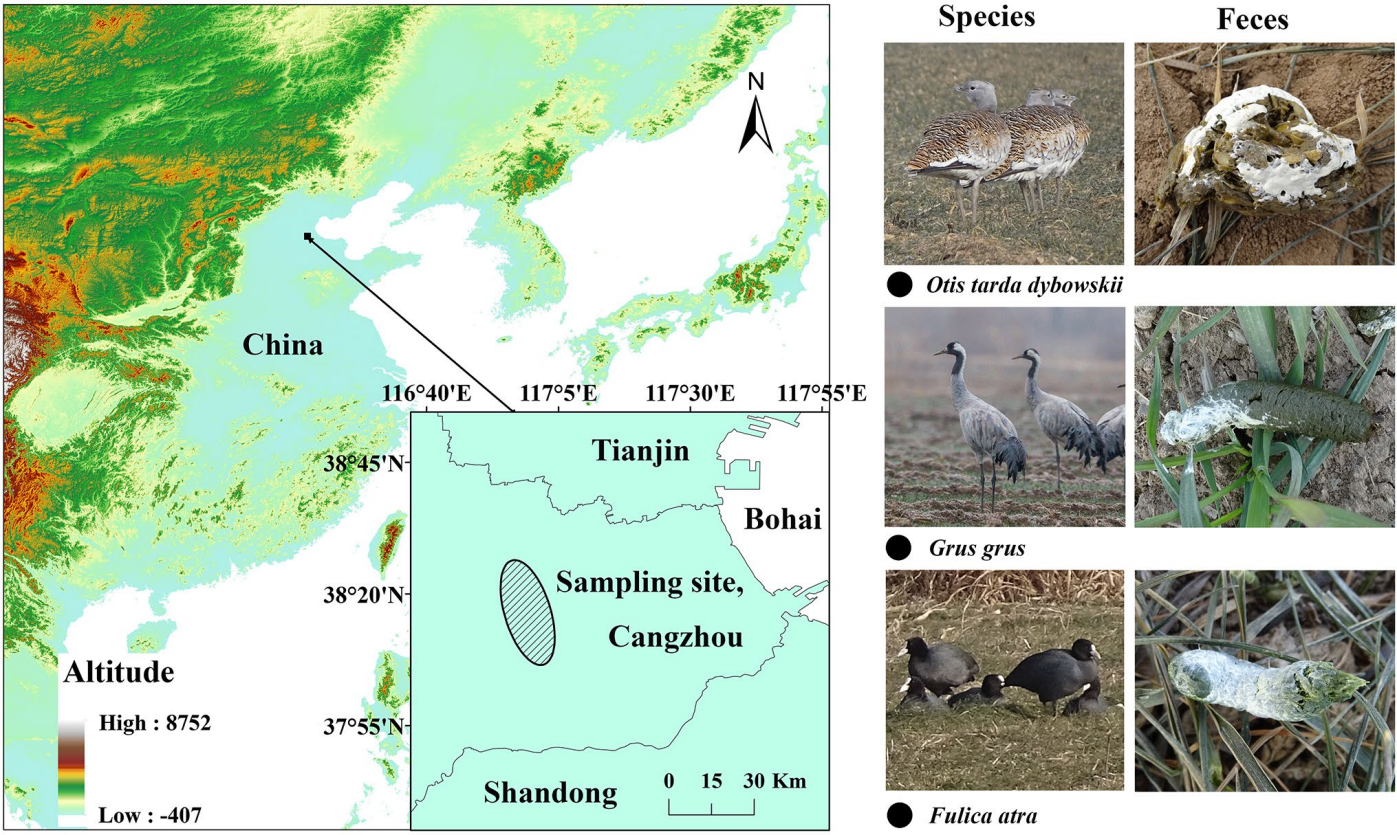
Fecal-sampling sites of the three wintering wild bird species.

We collected a total of 66 fresh fecal samples from the wild populations of the Great Bustard (OT 1–22, *n* = 22), Common Coot (FA 1–23, *n* = 23), and Common Crane (GG 1–21, *n* = 21) in the central-eastern part of the North China Plain (Figure 1). The North China Plain is the largest agricultural production area in China and usually adopts a double-cropping system consisting of wheat in the winter followed by maize in the summer (Wu et al., 2006; Wang et al., 2008), and the mean air temperature in this region is <0°C between late November and early March (Li et al., 2005).

According to our wild-bird survey records in the sampling area, the Common Coot is sedentary, and Great Bustard and Common Crane remain stable from November to February. All the samples were collected within 1 month during the winter, between 20 December 2020 and 20 January 2021. We tracked the three species by using monoculars (Zeiss, 22–65 × 85), selected populations with >20 individuals, determined that no other species were present in the sampling area, and searched for fresh samples as soon as the birds left. The minimum distance between two samples was maintained at 5 m to avoid collecting multiple samples from the same individual. The middle portions of the feces were sampled into 15 ml sterile centrifuge tubes by using sterile disposable forceps, transported in a −20°C portable freezer, and stored at −80°C before processing.

### Fecal-Sample DNA Extraction, Amplification, and Sequencing

The OMEGA-soil DNA kit (Omega Bio-Tek, United States) was used for the total-DNA extraction. The NanoDrop 2000 UV–vis spectrophotometer (NanoDrop Technologies, United States) was used to assess the concentration and purity of the final DNA sample. DNA quality was further examined *via* 1% agarose gel electrophoresis. Polymerase chain reaction (PCR) amplification of the V3-V4 hypervariable regions of the *16S rRNA* gene was then conducted on a thermocycler PCR system (GeneAmp® 9700, ABI, United States) using the pre-barcoded 338 F (5′-ACTCCTACGGGAGGCAGCAG-3) and 806 R (5′-GGACTACHVGGGTWTCTAAT-3′) primers (Mori et al., 2014). The cycling conditions were initial denaturation at 95°C for 3 min; followed by 27 cycles at 95°C for 30 s, 55°C for 30 s, and 72°C for 45 s; and final extension at 72°C for 10 min. The PCR reaction mix contained 4 μl of 5× FastPfu buffer, 2 μl of 2.5 mM dNTPs, 0.8 μl of 5 μM each primer, 0.4 μl FastPfu DNA polymerase, 10 ng DNA template, and double-distilled H_2_O in a total volume of 20 μl. The PCR products were electrophoresed on a 2% agarose gel, purified using the AxyPrep DNA Gel Extraction Kit (Axygen Biosciences, Union City, CA, United States), and quantified using the QuantiFluor™ -ST System (Promega, United States). Purified amplicons were pooled in equimolar concentrations and pair-end 2 × 300 bp sequencing was performed on an Illumina MiseqPE 300 platform by Majorbio Bio-Pharm Technology Co., Ltd. (Shanghai, China), according to the standard protocols.

### Data Processing and Analysis

Raw sequences were processed using the standard procedures of QIIME 2 (Bolyen et al., 2019). After demultiplexing, the resulting sequences were merged using FLASH v1.2.11 (Magoc and Salzberg, 2011) and quality-filtered using the Fastp v0.19.6 software (Chen et al., 2018). Then, DADA 2 was used to de-noise the high-quality sequences (Callahan et al., 2016), and each sequence was classified and annotated using the Naive Bayes classifier against the Silva database (SSU138) at a 70% confidence threshold. We removed chloroplastic and mitochondrial amplicon sequence variants (ASVs).

Rarefaction curves were subsequently plotted based on each sample’s observed richness (Sobs) to evaluate the sequencing efficiency. Mothur v1.30.2 was used to calculate alpha-diversity indices based on the ASVs, including Sobs, community evenness (Shannoneven), community diversity (Shannon), and community coverage (Good’s Coverage). The significance of alpha-diversity difference was evaluated using the Kruskal-Wallis test with multiple testing corrections (False Discovery Rate, FDR). The similarity in gut-microbiota composition among groups was based on the Bray–Curtis distance by using the principal coordinate analysis (PCoA). Statistical significance was assessed *via* Analysis of Similarities (ANOSIM) using 999 random permutations. The linear discriminant analysis (LDA) coupled with the effect size (LEfSe) based on the Kruskal-Wallis test was utilized to analyze the taxon, for which the relative abundance was significantly different among groups (*p* < 0.05, LDA value >4). The analysis was performed following the guide on the galaxy platform (Afgan and Baker, 2018). The hierarchical clustering of the samples was based on the Bray–Curtis distances by using the Unweighted Pair-group Method with Arithmetic Mean (UPGMA). The functions of the ASVs in each sample were analyzed using PICRUSt 2 set at the default parameters, by following the Kyoto Encyclopedia of Genes and Genomes (KEGG) database (Douglas et al., 2020). The KEGG level II pathways among different species were visualized using a hierarchical-clustering tree based on the Average clustering method and the Bray–Curtis distances.

To present the taxonomy of the hosts, a phylogenetic tree was constructed based on the consensus bird phylogenetic tool available at: http://www.birdtree.org (Jetz et al., 2012; Rubolini et al., 2015).

## Results

### Sequencing-Data Analysis and Microbiota Composition

After quality control and de-noising, a total of 2,315,700 valid sequences were produced from all the samples, ranging from 21,753 to 48,479 sequences per sample (Supplementary Table S1). To avoid statistical differences caused by different sequencing depths, we sub-sampled the original sequences according to the minimum number of reads (21,753 reads only), resulting in a total of 1,435,698 sequence reads. In total, 4,280 ASVs were identified and classified. The Great Bustard had 17 phyla, 27 classes, 78 orders, 119 families, and 219 genera; the Common Crane had 31 phyla, 83 classes, 189 orders, 320 families, and 622 genera; and the Common Coot had 21 phyla, 41 classes, 100 orders, 167 families, and 286 genera. The coverage of the end-use sequence data was evaluated using Good’s coverage and rarefaction curves analysis. The bacterial diversity per community was >99% (Supplementary Table S2), as calculated using Good’s coverage. The rarefaction curves provided a visual representation of the bacterial diversity coverage in each host (Supplemetary Figure S1). The microbial communities of all the samples were well represented.

**Figure 2:**
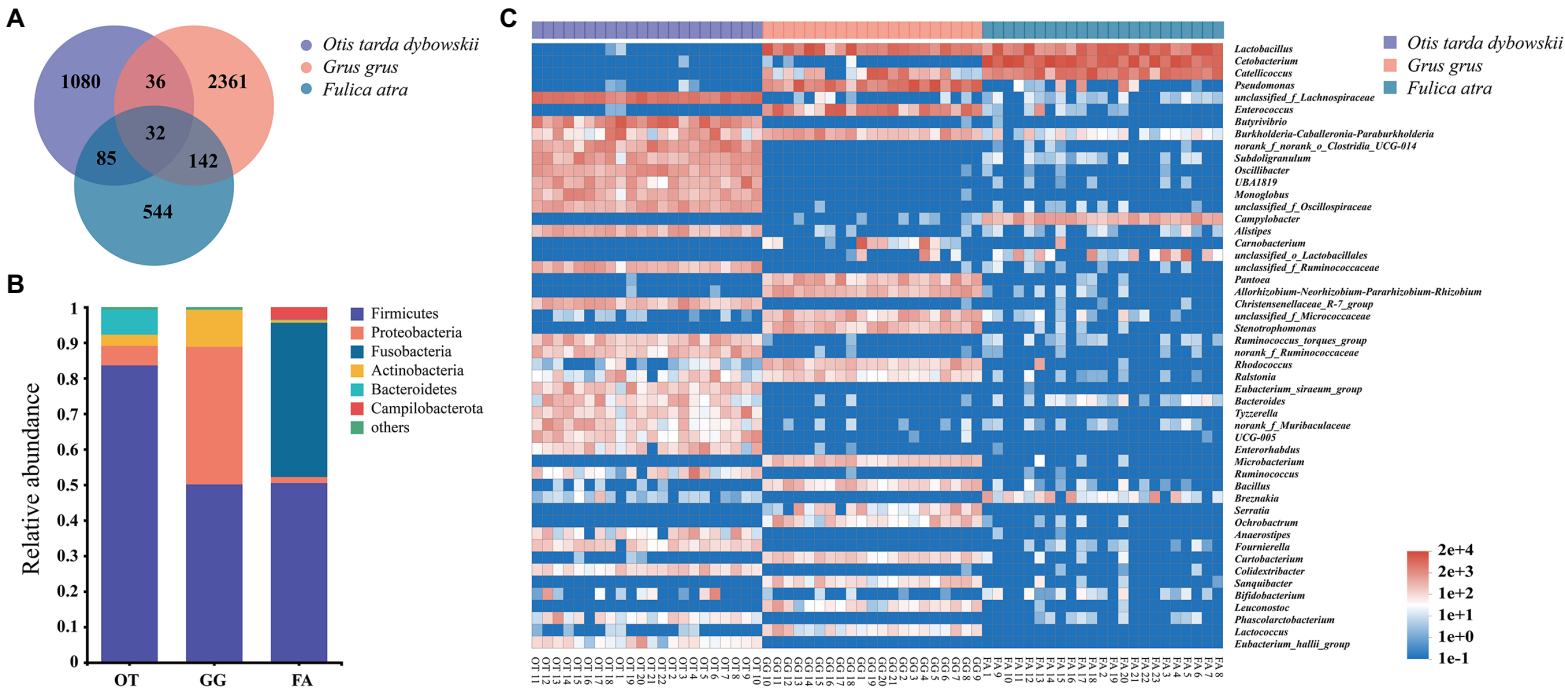
**(A)** The Venn diagram of the amplicon sequence variants (ASVs) in all the fecal samples. **(B)** Relative abundance of the microbial communities at the phylum level. The sequences that account for <1% were combined as “others.” OT, *Otis tarda dybowskii*; GG, *Grus grus*; and FA, *Fulica atra*. **(C)** Heatmap showing the microbiota compositions of the top 50 genera in total abundance across all the samples.

The microbial compositions of 66 samples were analyzed, and the mean relative microbial abundance was subsequently calculated at the phylum level. A total of 33 phyla were detected, and Firmicutes, Proteobacteria, Fusobacteria, Actinobacteria, Bacteroidetes, and Campilobacterota were the dominant phyla with relative abundance >1%. Firmicutes constituted the majority in the gut microbiota of all the three birds, descending in the order of the Great Bustard, Common Crane, and Common Coot (83.62%, 50.12%, and 50.52%, respectively; Figure 2B). At the genus level, a total of 759 genera were detected. We used the heatmap analysis to show the top 50 most abundant genera in all the samples as a whole, differences in microbiota composition were evident among the species (Figure 2C). For the Great Bustard, the genus with the highest relative abundance was found to be *unclassified- f - Lachnospiraceae*. *Lactobacillus* and *Cetobacterium* were the dominant microbiomes of the Common Crane and Common Coot, respectively. At the ASV level, the Venn diagram showed that only 0.75% of the ASVs were shared by the three species; the Great Bustard and Common Crane shared the lowest number of ASVs, whereas the Common Crane and Common Coot shared the highest number of ASVs (Figure 2A).

We assessed the three wild bird species for the presence of pathogenic microbes, and detected 13 potentially pathogenic genera (Supplementary Table S3). Of these genera, the Common Crane, Common Coot, and Great Bustard had 12, 6, and 3 genera, respectively. The 3 pathogenic genera detected in the bustard—*Escherichia-Shigella*, *Clostridium*, and *Helicobacter*—were common among the three hosts. The Common Crane and Common Coot shared 5 pathogenic genera. *Pasteurella* was only detected in the Common Coot *Staphylococcus*, *Vibrio*, *Macrococcus*, *Haemophilus*, and *Yersinia* were the endemic species of the Common Crane. Of the potential pathogenic genera, *Campylobacter* showed the highest relative abundance (3.27%), whereas the relative abundance of the other pathogenic genera remained low (<0.23%).

### Gut-Microbiota Diversity Analyses

**Figure 3:**
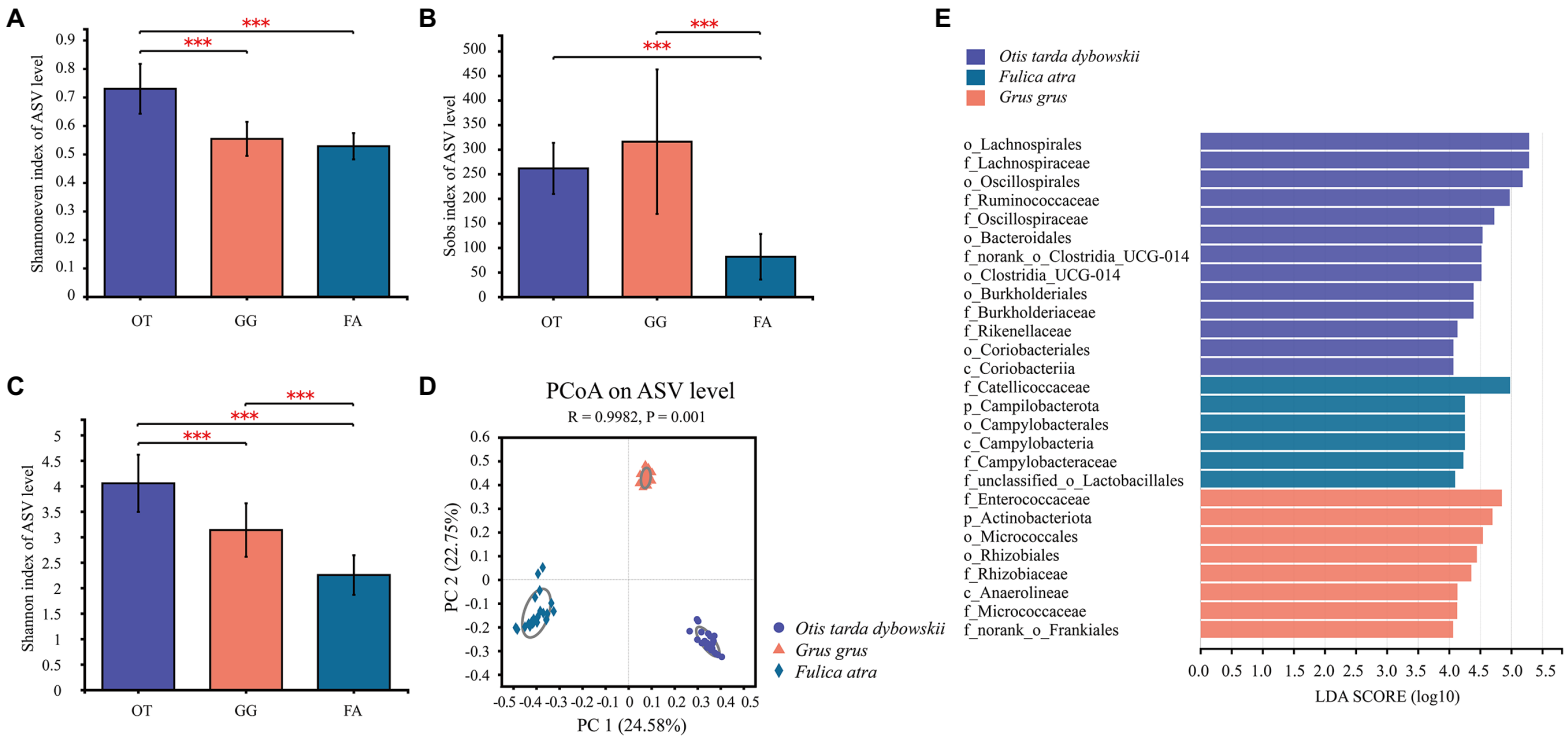
Boxplot showing the differences in alpha diversity among the three wintering wild bird species (^***^p < 0.001). **(A)** Shannoneven, **(B)** Sobs, and **(C)** Shannon indices. OT, *Otis tarda dybowskii*; GG, *Grus grus*; and FA, *Fulica atra*. **(D)** PCoA of the gut microbial communities of the three bird species according to the Bray–Curtis distances. The differences among the three groups were assessed using the Analysis of Similarities (ANOSIM) with 999 permutations. **(E)** LEfSe analysis on the gut microbial biomarkers of the three bird species (LDA > 4, *p* < 0.05).

Comparative analysis of alpha-diversity indices (mean ± SE) revealed the differences in gut microbial community diversity among the three wild bird species living in sympatric habitats (Supplementary Table S4). Community richness (Sobs) based on ASVs revealed that the indices of the Great Bustard (261.3 ± 52.05) and Common Crane (315.7 ± 147.1) were significantly higher than that of the Common Coot (81.48 ± 46.41, *p* < 0.001), and there was no significant difference between the Great Bustard and Common Crane (Figure 3B). Additionally, the community evenness (Shannoneven) of the Great Bustard (0.73 ± 0.087) was significantly greater than the Common Crane (0.55 ± 0.06, *p* < 0.001) and Common Coot (0.53 ± 0.046, *p* < 0.001), and there was no significant difference between the Common Crane and Common Coot (Figure 3A). All the three host species were significantly different from each other in community diversity (Shannon; *p* < 0.001), with the highest index calculated for the Great Bustard (4.05 ± 0.56), and the lowest for the Common Coot (2.25 ± 0.39; Figure 3C).

To visualize the variation in bacterial community composition among the three bird species, PCoA based on the Bray–Curtis distances revealed that bacterial community-independent aggregation was characterized by host species; the largest amount of variation was 24.58% (Figure 3D). This result suggests that each species harbored a unique gut microbial community composition. Analysis of similarity (ANOSIM) demonstrated that there were significant differences in gut microbial community composition among the hosts (*R* = 0.9982, *p* = 0.001) and that each species had a unique microbial community composition (Supplementary Table S5). *Via* LEfSe analysis, we identified the specific taxa of the gut microbes and screened the representative taxa that significantly differed among the hosts. Consequently, 13 features were found differentially abundant in the Great Bustard. Of them, six, six, and one were identified at the family, order, and class levels, respectively. In the Common Crane, eight features were differentially abundant, and of them, four, two, one, and one were identified at the family, order, class, and phylum levels, respectively. In the Common Coot, six features (three, one, one, and one, at the family, order, class, and phylum levels, respectively) were differentially abundant (Figure 3E).

### Host Phylogenetic Tree, Gut-Microbiota Hierarchical Clustering, and Functional Predictions

**Figure 4:**
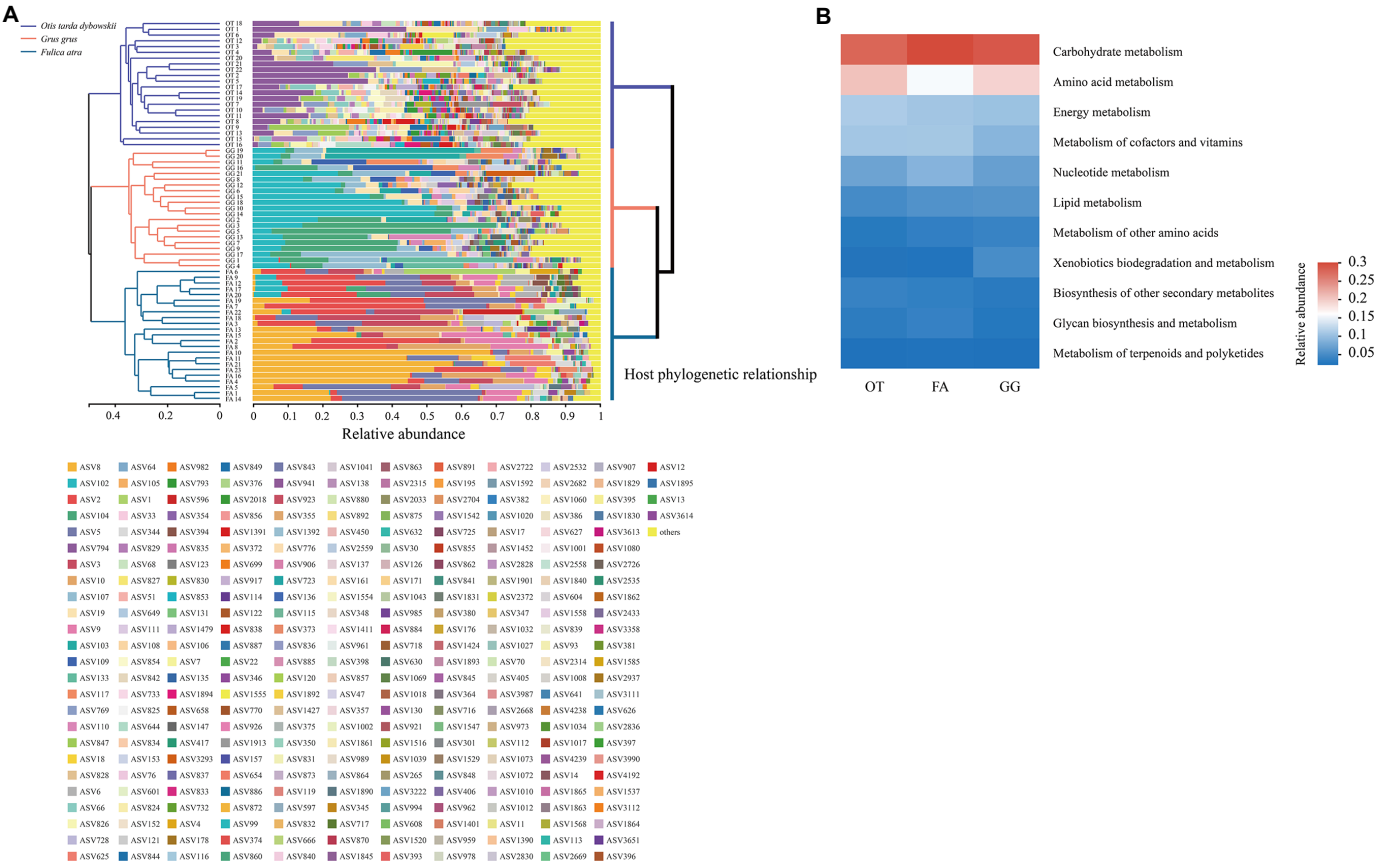
**(A)** Left: The Unweighted Pair-group Method with Arithmetic Mean (UPGMA) based on Bray–Curtis distances showed the hierarchical clustering of the gut microbiota. Stacked barplot showed the ASVs in each sample, and the ASVs with relative abundance <0.01 were combined as “others.” Right: Host phylogenetic tree constructed using the brid tree tools. **(B)** Relative abundance of the metabolic pathway categories in the gut microbiota of the three wild bird species. OT, *Otis tarda dybowskii*; GG, *Grus grus*; and FA, *Fulica atra*.

Hierarchical clustering analysis (UPGMA) revealed that samples from the same host strictly clustered together even though there were large individual differences among them (Figure 4A). The host phylogenetic tree showed that the Common Crane and Common Coot had a shorter evolutionary distance than the Great Bustard, consistent with their gut microbial hierarchical clustering topology (Figure 4A). A total of 46 KEGG level II pathways were identified in the three bird species, with high-abundance functions mainly focused on carbohydrate metabolism, amino acid metabolism, membrane transport, energy metabolism, and metabolism of cofactors and vitamins. The hierarchical clustering tree of the 46 KEGG level II pathway categories showed that the abundance of KEGG functional pathways was not strictly clustered by the host, in contrast to the UPGMA analysis of gut microbial community composition (Supplemetary Figure S2). We used a heatmap to demonstrate the enriched major metabolic pathways contained in metabolism, and carbohydrate metabolism was identified as the most important metabolic pathway in the gut microbiota of the three bird species (Figure 4B).

## Discussion

This is the first study to present the gut microbiota of multiple sympatric farmland-dependent birds, and the first report on the gut microbiota of the Common Coot. Under natural conditions, one cannot precisely control the environment visited by the hosts or the food consumed by them. Therefore, it is difficult to disentangle the individual effects of environmental and genetic factors on the gut microbiota. Several studies have identified dietary factors influencing the gut microbiota; however, in these studies, there were significant differences in dietary composition among the individuals within the same species (Bodawatta et al., 2022b), and broad dietary classifications (e.g., herbivorous, carnivorous, and omnivorous) were more significant than the variations in diet (Hird et al., 2015). In the winter, farmland-dependent birds have to become herbivory, and thus, our study species share the same dietary factors. Additionally, species sharing a large habitat are more conducive to cross-species gut microbial comparisons than those living in different habitats.

In our study, the gut microbiota of three wild bird species were found to mainly comprise six phyla, including Firmicutes, Proteobacteria, Fusobacteria, Actinobacteria, Bacteroidetes, and Campilobacterota, consistent with studies on other birds, such as the Greylag Geese (*Anser anser*; Wang et al., 2018), Bengalese Finch (*Lonchura striata domestica*) and Zebra Finch (*Taeniopygia guttata*; Maraci et al., 2021), Hooded Crane (*Grus monacha*; Fu et al., 2020), and Whooper Swan (*Cygnus cygnus*; Wang et al., 2021). Firmicutes, the most dominant species among the three hosts, generally dominate the gut microbiota in all animals and play an important role in maintaining gut homeostasis and assisting digestion. Several previous studies have demonstrated that many members of Firmicutes and Bacteroidetes can express carbohydrate-active enzymes, helping the host to hydrolyze and utilize carbohydrates (Kaoutari et al., 2013). Dietary cornstarch supplements significantly increase the overall abundance of Firmicutes and Bacteroidetes in the poultry cecum (Zhang et al., 2020). In mid-to-high latitude wintering grounds, the Great Bustard, Common Crane, and Common Coot have to become herbivory due to the low-temperature stress. As habitats, these birds prefer farmlands near rivers and forage for grain seeds and the vegetative parts of the plants scattered in stubble or winter wheat fields (Mouronval et al., 2007; Harris and Mirande, 2013; Liu et al., 2018). Our results from the functional predictions of the gut microbiota at the KEGG level II pathways, with the carbohydrate metabolic pathway being the most enriched metabolic pathway, support these previous findings. The high proportion of Firmicutes in the three hosts presumably helps the digestion of complex polymers such as polysaccharides and cellulose in plant-rich diets and improves the energy conversion efficiency.

In several recent comparative interspecies studies, researchers have noted that gut morphology has an important influence on the gut microbiota (Amato et al., 2019; Lee et al., 2020). Compared with long digestive tracts, short ones have less food retention time, and their physiological environment is less stable (Bodawatta et al., 2022a). Gut retention time is strongly associated with the body mass of the host (Jackson, 1992; Wotton and Kelly, 2012). In our study, the body masses of the three hosts were significantly different and in the order of the Great Bustard > the Common Crane > the Common Coot. The Shannon diversity difference test showed significant differences in gut microbial composition among these three hosts (*p* < 0.001), with the highest community diversity in the Great Bustard and the lowest in the Common Coot. Gut microbial diversity was found to remain positively correlated with the gut retention time of the host. In addition, the Great Bustard, Common Crane, and Common Coot all have well-developed ceca, which increases the retention time of food in the gut and promotes the stability of gut symbionts (Fanke et al., 2011; Crompton and Nesheim, 2016; Li et al., 2017). *Via* PCoA analysis coupled with ANOSIM statistical test, we consistently revealed that these three sympatric wild birds have significantly different gut microbiota. The Venn diagram indicated that <1% of the ASVs are shared among the hosts. The above results indicate that the gut microbiota of different host species do not undergo adaptive convergence and instead maintain relatively independent structures under similar environmental conditions. Host-species–specific differences and phylogenetic correlations among microbiota are considered to be evidence for genetic factors determining the gut microbiota (Ley et al., 2008). Thus, we analyzed the relationship between the host phylogeny and gut microbiota. The taxonomic positions of the three host species were well resolved, with the Common Crane and Common Coot belonging to the same order (Gruiformes) and being closer to the common phylogenetic ancestor than the Great Bustard (Otidiformes). The UPGMA analysis results demonstrated that although there is a certain individual variation in the microbial composition of each sample, all the samples are strictly clustered with the host species. The gut microbiota and host phylogeny both had similar tree topologies, indicating that genetic factors drive the host gut microbiota.

Interestingly, we also detected 13 pathogenic genera in these three overwintering farmland-dependent birds, and *Escherichia-Shigella*, *Clostridium*, and *Helicobacter* were detected in all the three hosts. Studies have shown that ammonia induces lung injury in broiler chickens by activating the NLRP3 inflammasome *via* gut-resident *Escherichia-Shigella* (Liu et al., 2020). The increase in the relative abundance of gut-resident *Escherichia-Shigella* affects the meat yield from broiler chickens (Rubio et al., 2015; Han et al., 2021). *Clostridium perfringens* in the gut can cause necrotic enteritis, which is considered the most clinically dramatic bacterial enteric disease of poultry (Cooper and Songer, 2009). *Helicobacter pullorum* causes 33.3% mortality in chickens, and surviving individuals show symptoms of diarrhea, poor growth, and poor transformation rate (Hassan et al., 2014). In addition, we found that the Common Crane and Common Coot share two other pathogenic genera, *Mycobacterium* and *Campylobacter*. Bird tuberculosis, caused by *Mycobacterium avium* or *Mycobacterium genavense* is one of the most important diseases affecting poultry and pet birds, with clinical manifestations including emaciation, depression, and diarrhea, alongside marked atrophy of the breast muscle (Dhama et al., 2011). The effect of *Clostridium jejuni* on chicken intestinal function has been shown to interfere with chicken performance and welfare (Awad et al., 2018). In our study, the detection rate and relative abundance of pathogenic genera in the hosts were at low levels, but it cannot be ruled out that the shared habitat during the wintering period may lead to outbreaks of periodic infections among sympatric migratory birds. Usage of non-invasive methods to monitor the changes in pathogenic species in wild bird fecal samples has important implications for the conservation of endangered species.

## Conclusion

Our comparative study on bird gut microbial communities is one of the few studies on sympatric terrestrial wild birds and the first to describe the gut microbiota of a wild population of the Common Coot. After simplifying the complex effects of the various influencing factors of natural conditions, it was found that the gut microbiota of different hosts do not undergo adaptive convergence, maintain relatively independent structures, and respond to the evolutionary relationships of the hosts. In addition, these host species carry various pathogenic microbes. Due to the limitations of the sequencing technology, we did not discuss the microbiota functions of the three species in depth. In future work, we will integrate metagenomics and targeted metabolomics to deeply explore the functions of the gut microbiota, which may improve wildlife conservation and captive-management strategies.

## Data Availability Statement

The data presented in the study are deposited the NCBI database (Accession numbers: PRJNA 720495, PRJNA 818137, PRJNA818147).

## Author Contributions

ZL collected samples, analyzed the data, prepared figures and tables, authored or reviewed drafts of the paper, and approved the final draft. DM collected samples and provided basic data about the wintering habitat of the three wild birds. SL, MW, and CW analyzed the data. JL conceived and designed the experiments, authored or reviewed drafts of the paper, and approved the final draft. All authors contributed to the article and approved the submitted version.

## Funding

This work was supported in part by the Postgraduate Innovation Foundation of Hebei (grant number: CXZZBS2021062), and in part by the S&T Program of Hebei (grant number: 19K56233D).

## Conflict of Interest

The authors declare that the research was conducted in the absence of any commercial or financial relationships that could be construed as a potential conflict of interest.

## Publisher’s Note

All claims expressed in this article are solely those of the authors and do not necessarily represent those of their affiliated organizations, or those of the publisher, the editors and the reviewers. Any product that may be evaluated in this article, or claim that may be made by its manufacturer, is not guaranteed or endorsed by the publisher.

## Supplementary Material

The Supplementary Material for this article can be found online at: https://www.frontiersin.org/articles/10.3389/fmicb. 2022.905668/full#supplementary-material

## References

[ref1] Afgan E. Baker D. (2018). The galaxy platform for accessible, reproducible and collaborative biomedical analyses: 2018 update. Nucleic Acids Res. 46, W537–W544. doi: 10.1093/nar/gky379, PMID: 29790989 PMC6030816

[ref2] Alonso J. C. (2015). The great bustard: past, present and future of a globally threatened species. Ornis Hung. 22, 1–13. doi: 10.2478/orhu-2014-0014

[ref3] Alonso J. C. Bautista L. M. Alonso J. A. (2004). Family-based territoriality vs flocking in wintering common cranes *Grus grus*. J. Avian Biol. 35, 434–444. doi: 10.1111/j.0908-8857.2004.03290.x

[ref4] Alonso J. C. Palacin C. (2010). The world status and population trends of the great bustard (*Otis tarda*): 2010 update. Chinese Birds 1, 141–147. doi: 10.5122/cbirds.2010.0007

[ref5] Amato K. R. Sanders J. G. Song S. J. Nute M. Metcalf J. L. Thompson L. R.. (2019). Evolutionary trends in host physiology outweigh dietary niche in structuring primate gut microbiomes. ISME J. 13, 576–587. doi: 10.1038/s41396-018-0175-0, PMID: 29995839 PMC6461848

[ref6] Awad W. A. Hess C. Hess M. (2018). Re-thinking the chicken *–Campylobacter jejuni* interaction: a review. Avian Pathol. 47, 352–363. doi: 10.1080/03079457.2018.1475724, PMID: 29764197

[ref7] Bodawatta K. H. Hird S. M. Grond K. Poulsen M. Jønsson K. A. (2022a). Avian gut microbiomes taking flight. Trends Microbiol. 30, 268–280. doi: 10.1016/j.tim.2021.07.003, PMID: 34393028

[ref8] Bodawatta K. H. Klečková I. Klečka J. Pužejová K. Koane B. Poulsen M.. (2022b). Specific gut bacterial responses to natural diets of tropical birds. Sci. Rep. 12:713. doi: 10.1038/s41598-022-04808-9, PMID: 35027664 PMC8758760

[ref9] Bodawatta K. H. Koane B. Maiah G. Sam K. Poulsen M. Jønsson K. A. (2021). Species-specific but not phylosymbiotic gut microbiomes of new Guinean passerine birds are shaped by diet and flight-associated gut modifications. Proc. R. Soc. B 288:20210446. doi: 10.1098/rspb.2021.0446, PMID: 33878920 PMC8059580

[ref10] Bolyen E. Rideout J. R. Dillon M. R. Bokulich N. A. Abnet C. C. Al-Ghalith G. A.. (2019). Reproducible, interactive, scalable and extensible microbiome data science using QIIME 2. Nat. Biotechnol. 37, 852–857. doi: 10.1038/s41587-019-0209-9, PMID: 31341288 PMC7015180

[ref11] Callahan B. J. McMurdie P. J. Rosen M. J. Han A. W. Johnson A. J. A. Holmes S. P. (2016). DADA2: high-resolution sample inference from Illumina amplicon data. Nat. Methods 13, 581–583. doi: 10.1038/nmeth.3869, PMID: 27214047 PMC4927377

[ref12] Chen S. Zhou Y. Chen Y. Gu J. (2018). Fastp: an ultra-fast all-in-one FASTQ preprocessor. Bioinformatics 34, i884–i890. doi: 10.1093/bioinformatics/bty560, PMID: 30423086 PMC6129281

[ref13] Collar N. J. Baral H. S. Batbayar N. Bhardwaj G. S. Brahma N. Burnside R. J.. (2017). Averting the extinction of bustards in Asia. Forktail 33, 1–26.

[ref14] Cooper K. K. Songer J. G. (2009). Necrotic enteritis in chickens: a paradigm of enteric infection by *Clostridium perfringens* type A. Anaerobe 15, 55–60. doi: 10.1016/j.anaerobe.2009.01.006, PMID: 19186215

[ref15] Cramp S. Simmons K. E. L. (1980). Handbook of the Birds of Europe the Middle East and North Africa. The Birds of the Western Palearctic, Vol. II. Oxford University Press, Oxford.

[ref16] Crompton D. Nesheim M. (2016). Survey of the Avian Alimentary Tract. Internet-First University Press, Ithaca, NY.

[ref17] Dhama K. Mahendran M. Tiwari R. Dayal Singh S. Kumar D. Singh S.. (2011). Tuberculosis in birds: insights into the *Mycobacterium avium* infections. Vet. Med. Int. 2011, 1–14. doi: 10.4061/2011/712369, PMID: 21776352 PMC3135220

[ref18] Douglas G. M. Maffei V. J. Zaneveld J. R. (2020). PICRUSt2 for prediction of metagenome functions. Nat. Biotechnol. 38, 685–688. doi: 10.1038/s41587-020-0548-6, PMID: 32483366 PMC7365738

[ref19] Dunning J. B Jr. (2008). CRC Handbook of Avian Body Masses, 2nd ed. Boca Raton, FL: CRC Press, Florida, USA.

[ref20] English M. D. Robertson G. J. Peck L. E. Mallory M. L. (2017). Agricultural food resources and the foraging ecologies of American black ducks (*Anas rubripes*) and mallards (*Anas platyrhynchos*) at the northern limits of their winter ranges. Urban Ecosyst. 20, 1311–1318. doi: 10.1007/s11252-017-0683-0

[ref21] Fanke J. Wibbelt G. Krone O. (2011). Mortality factors and diseases in free-range Eurasian cranes (*Grus grus*) in Germany. J. Wildl. Dis. 47, 627–637. doi: 10.7589/0090-3558-47.3.627, PMID: 21719827

[ref22] Faragó S. (2019). Spectrum of plant and animal diet of European great bustard (*Otis tarda tarda*) – an overview. Ornis Hungarica 27, 62–84. doi: 10.2478/orhu-2019-0004

[ref23] Fu R. Xiang X. Dong Y. Cheng L. Zhou L. (2020). Comparing the intestinal bacterial communies of sympatric wintering hooded crane (*Grus monacha*) and domestic goose (*Anser anser* domesticus). Avian Res. 11:13. doi: 10.1186/s40657-020-00195-9

[ref24] Gillingham M. A. F. Béchet A. Cézilly F. Wilhelm K. Rendón-Martos M. Borghesi F.. (2019). Offspring microbiomes differ across breeding sites in a panmictic species. Front. Microbiol. 10:35. doi: 10.3389/fmicb.2019.00035, PMID: 30787910 PMC6372503

[ref25] Grond K. Ryu H. Baker A. J. Domingo J. W. S. Buehler D. M. (2014). Gastro-intestinal microbiota of two migratory shorebird species during spring migration staging in Delaware Bay, USA. J. Ornithol. 155, 969–977. doi: 10.1007/s10336-014-1083-3

[ref26] Grond K. Sandercock B. K. Jumpponen A. Zeglin L. H. (2018). The avian gut microbiota: community, physiology and function in wild birds. J. Avian Biol. 49:e01788. doi: 10.1111/jav.01788

[ref27] Han H. Zhou Y. Liu Q. Wang G. Feng J. Zhang M. (2021). Effects of ammonia on gut microbiota and growth performance of broiler chickens. Animals 11:1716. doi: 10.3390/ani11061716, PMID: 34201291 PMC8228959

[ref28] Harris J. Mirande C. (2013). A global overview of cranes: status, threats and conservation priorities. Chinese Birds 4, 189–209. doi: 10.5122/cbirds.2013.0025

[ref29] Harris J. Su L. Higuchi H. Ueta M. Zhang Z. Zhang Z.. (2000). Migratory stopover and wintering locations in eastern China used by White-naped Cranes *Grus vipio* and Hooded Cranes *G. monacha*, as determined by satellite tracking. Forktail 16, 93–99.

[ref30] Harrison C. (1982). Atlas of the birds of the Western Palaearctic. HarperCollins, New York, NY.

[ref31] Hassan A. K. Shahata M. A. Refaie E. M. Ibrahim R. S. (2014). Pathogenicity testing and antimicrobial susceptibility of *Helicobacter pullorum* isolates from chicken origin. Int. J. Vet. Sci. Med. 2, 72–77. doi: 10.1016/j.ijvsm.2013.12.001

[ref32] Hird S. M. Sánchez C. Carstens B. C. Brumfield R. T. (2015). Comparative gut microbiota of 59 Neotropical bird species. Front. Microbiol. 6:1403. doi: 10.3389/fmicb.2015.01403, PMID: 26733954 PMC4685052

[ref33] Jackson S. (1992). Do seabird gut sizes and mean retention times reflect adaptation to diet and foraging method? Physiol. Zool. 65, 674–697. doi: 10.1086/physzool.65.3.30157976

[ref34] Jetz W. Thomas G. H. Joy J. B. Hartmann K. Mooers A. O. (2012). The global diversity of birds in space and time. Nature 491, 444–448. doi: 10.1038/nature11631, PMID: 23123857

[ref35] Johnsgard P. A. (1983). Cranes of the World: Eurasian Crane (*Grus grus*). Bloomington, USA: Indiana University Press. Available at: https://digitalcommons.unl.edu/bioscicranes/17

[ref36] Kaoutari A. E. Armougom F. Gordon J. I. Raoult D. Henrissat B. (2013). The abundance and variety of carbohydrate-active enzymes in the human gut microbiota. Nat. Rev. Microbiol. 11, 497–504. doi: 10.1038/nrmicro3050, PMID: 23748339

[ref37] Kessler A. E. Batbayar N. Natsagdorj T. Batsuur’ D. Smith A. T. (2013). Satellite telemetry reveals long-distance migration in the Asian great bustard *Otis tarda dybowskii*. J. Avian Biol. 44, 311–320. doi: 10.1111/j.1600-048X.2013.00072.x

[ref38] Laviad-Shitrit S. Izhaki I. Lalzar M. Halpern M. (2019). Comparative analysis of intestine microbiota of four wild waterbird species. Front. Microbiol. 10:1911. doi: 10.3389/fmicb.2019.01911, PMID: 31481943 PMC6711360

[ref39] Lee C. Y. Peralta-Sánchez J. M. Martínez-Bueno M. Møller A. P. Rabelo-Ruiz M. Zamora-Muñoz C.. (2020). The gut microbiota of brood parasite and host nestlings reared within the same environment: disentangling genetic and environmental effects. ISME J. 14, 2691–2702. doi: 10.1038/s41396-020-0719-y, PMID: 32681160 PMC7784868

[ref40] Leito A. Truu J. Leivits A. Ojaste I. (2003). Changes in distribution and numbers of the breeding population of the common crane *Grus grus* in Estonia. Ornis Fenn. 80, 159–171.

[ref41] Lewis W. B. Moore F. R. Wang S. (2017). Changes in gut microbiota of migratory passerines during stopover after crossing an ecological barrier. Auk 134, 137–145. doi: 10.1642/AUK-16-120.1

[ref42] Ley R. E. Hamady M. Lozupone C. Turnbaugh P. J. Ramey R. R. Bircher J. S.. (2008). Evolution of mammals and their gut microbes. Science 320, 1647–1651. doi: 10.1126/science.1155725, PMID: 18497261 PMC2649005

[ref43] Li L. Hu R. Huang J. Bürgi M. Zhu Z. Zhong J.. (2020). A farmland biodiversity strategy is needed for China. Nat. Ecol. Evol. 4, 772–774. doi: 10.1038/s41559-020-1161-2, PMID: 32221478

[ref44] Li J. Inanaga S. Li Z. Eneji A. E. (2005). Optimizing irrigation scheduling for winter wheat in the North China Plain. Agric. Water Manag. 76, 8–23. doi: 10.1016/j.agwat.2005.01.006

[ref45] Li C. Liu Y. Gong M. Zheng C. Zhang C. Li H.. (2021). Diet-induced microbiome shifts of sympatric overwintering birds. Appl. Microbiol. Biot 105, 5993–6005. doi: 10.1007/s00253-021-11448-y, PMID: 34272578

[ref46] Li W. Liu Y. Tian X. (2017). Scanning electron microscopic observations of the digestive canal of the great bustard (*Otis tarda*). Avian Biol. Res. 10, 190–195. doi: 10.3184/175815617X14967389842918

[ref47] Liu G. Shafer A. B. A. Hu X. Li L. Ning Y. Gong M.. (2018). Meta-barcoding insights into the spatial and temporal dietary patterns of the threatened Asian Great Bustard (*Otis tarda dybowskii*) with potential implications for diverging migratory strategies. Ecol. Evol. 8, 1736–1745. doi: 10.1002/ece3.3791, PMID: 29435248 PMC5792609

[ref48] Liu Q. X. Zhou Y. Li X. M. Ma D. D. Xing S. Feng J. H.. (2020). Ammonia induce lung tissue injury in broilers by activating NLRP3 inflammasome via *Escherichia/Shigella*. Poult. Sci. 99, 3402–3410. doi: 10.1016/j.psj.2020.03.019, PMID: 32616234 PMC7597683

[ref49] Lu Z. Li S. Li H. Wang Z. Meng D. Liu J. (2021a). The gut microbiota of wild wintering great bustard (*Otis tarda dybowskii*): survey data from two consecutive years. PeerJ 9:e12562. doi: 10.7717/peerj.12562, PMID: 34909281 PMC8641483

[ref50] Lu Z. Zhai Y. Meng D. Kou G. Li H. Liu J. (2021b). Predicting the potential distribution of wintering Asian Great Bustard (*Otis tarda dybowskii*) in China: conservation implications. Glob. Ecol. Conserv. 31:e01817. doi: 10.1016/j.gecco.2021.e01817

[ref51] Magoc T. Salzberg S. L. (2011). FLASH: fast length adjustment of short reads to improve genome assemblies. Bioinformatics 27, 2957–2963. doi: 10.1093/bioinformatics/btr507, PMID: 21903629 PMC3198573

[ref52] Maraci Ö. Antonatou-Papaioannou A. Jünemann S. Castillo-Gutiérrez O. Busche T. Kalinowski J.. (2021). The gut microbial composition is species-specific and individual-specific in two species of estrildid finches, the Bengalese finch and the Zebra finch. Front. Microbiol. 12:619141. doi: 10.3389/fmicb.2021.619141, PMID: 33679641 PMC7933042

[ref53] Mi C. Falk H. Guo Y. (2016). Climate envelope predictions indicate an enlarged suitable wintering distribution for Great Bustards (*Otis tarda dybowskii*) in China for the 21st century. Peerj 4:e1630. doi: 10.7717/peerj.1630, PMID: 26855870 PMC4741084

[ref54] Mi C. Huettmann F. Sun R. Guo Y. (2017). Combining occurrence and abundance distribution models for the conservation of the Great Bustard. Peerj 5:e4160. doi: 10.7717/peerj.4160, PMID: 29255652 PMC5732545

[ref55] Mori H. Maruyama F. Kato H. Toyoda A. Dozono A. Ohtsubo Y.. (2014). Design and experimental application of a novel non-degenerate universal primer set that amplifies prokaryotic 16S rRNA genes with a low possibility to amplify eukaryotic rRNA genes. DNA Res. 21, 217–227. doi: 10.1093/dnares/dst052, PMID: 24277737 PMC3989492

[ref56] Mouronval J. B. Guillemain M. Canny A. Poirier F. (2007). Diet of non-breeding wildfowl Anatidae and Coot *Fulica atra* on the Perthois gravel pits, northeast France. Wild 57, 68–97.

[ref57] Murray M. H. Lankau E. W. Kidd A. D. Welch C. N. Ellison T. Adams H. C.. (2020). Gut microbiome shifts with urbanization and potentially facilitates a zoonotic pathogen in a wading bird. PLoS One 15:e0220926. doi: 10.1371/journal.pone.0220926, PMID: 32134945 PMC7058277

[ref58] Ojaste I. Leito A. Suorsa P. Hedenström A. Sepp K. Leivits M.. (2020). From northern Europe to Ethiopia: long-distance migration of Common Cranes (*Grus grus*). Ornis Fenn. 97, 12–25.

[ref59] Pearce D. S. Hoover B. A. Jennings S. Nevitt G. A. Docherty K. M. (2017). Morphological and genetic factors shape the microbiome of a seabird species (*Oceanodroma leucorhoa*) more than environmental and social factors. Microbiome 5:146. doi: 10.1186/s40168-017-0365-4, PMID: 29084611 PMC5663041

[ref60] Perrow M. R. Schutten J. H. Howes J. R. Holzer T. Madgwick F. J. Jowitt A. J. D. (1997). “Interactions between coot (*Fulica atra*) and submerged macrophytes: the role of birds in the restoration process,” in Shallow Lakes. eds. Kufel L. Prejs A. Rybak J. I. (Dordrecht, NL: Springer).

[ref61] Phillips J. N. Berlow M. Derryberry E. P. (2018). The effects of landscape urbanization on the gut microbiome: an exploration into the gut of urban and rural White-Crowned Sparrows. Front. Ecol. Evol. 6:148. doi: 10.3389/fevo.2018.00148

[ref62] Randler C. (2006). Disturbances by dog barking increase vigilance in coots *Fulica atra*. Eur J. Wildlife Res. 52, 265–270. doi: 10.1007/s10344-006-0049-z

[ref63] Rubio L. A. Peinado M. J. Ruiz R. Suárez-Pereira E. Ortiz Mellet C. García Fernández J. M. (2015). Correlations between changes in intestinal microbiota composition and performance parameters in broiler chickens. J. Anim. Physiol. Anim. Nutr. 99, 418–423. doi: 10.1111/jpn.12256, PMID: 25266875

[ref64] Rubolini D. Liker A. Garamszegi L. Z. Møller A. P. Saino N. (2015). Using the BirdTree.org website to obtain robust phylogenies for avian comparative studies: a primer. Curr. Zool. 61, 959–965. doi: 10.1093/czoolo/61.6.959 32256531 PMC7098689

[ref65] Ryu H. Grond K. Verheijen B. Elk M. Buehler D. M. Santo Domingo J. W. (2014). Intestinal microbiota and species diversity of *Campylobacter* and *Helicobacter* spp. in migrating shorebirds in Delaware Bay. Appl. Environ. Microb. 80, 1838–1847. doi: 10.1128/AEM.03793-13, PMID: 24413599 PMC3957654

[ref66] San Juan P. A. Hendershot J. N. Daily G. C. Fukami T. (2020). Land-use change has host-specific influences on avian gut microbiomes. ISME J. 14, 318–321. doi: 10.1038/s41396-019-0535-4, PMID: 31624349 PMC6908588

[ref67] Song S. J. Sanders J. G. Delsuc F. Metcalf J. Amato K. Taylor M. W.. (2020). Comparative analyses of vertebrate gut microbiomes reveal convergence between birds and bats. mBio 11, e02901–e02919. doi: 10.1128/mBio.02901-19, PMID: 31911491 PMC6946802

[ref68] Teyssier A. Rouffaer L. O. Saleh Hudin N. Strubbe D. Matthysen E. Lens L.. (2018). Inside the guts of the city: urban-induced alterations of the gut microbiota in a wild passerine. Sci. Total Environ. 612, 1276–1286. doi: 10.1016/j.scitotenv.2017.09.035, PMID: 28898933

[ref69] Wang W. Huang S. Yang L. Zhang G. (2021). Comparative analysis of the fecal bacterial microbiota of wintering Whooper Swans (*Cygnus Cygnus*). Front. Vet. Sci. 8:670645. doi: 10.3389/fvets.2021.670645, PMID: 34322532 PMC8310996

[ref70] Wang W. Liu Y. Yang Y. Wang A. Sharshov K. Li Y.. (2018). Comparative analyses of the gut microbiota among three different wild geese species in the genus Anser. J. Basic Microbiol. 58, 543–553. doi: 10.1002/jobm.201800060, PMID: 29668076

[ref71] Wang E. Yu Q. Wu D. Xia J. (2008). Climate, agricultural production and hydrological balance in the North China Plain. Int. J. Climatol. 28, 1959–1970. doi: 10.1002/joc.1677

[ref72] Wotton D. M. Kelly D. (2012). Do larger frugivores move seeds further? Body size, seed dispersal distance, and a case study of a large, sedentary pigeon. J. Biogeogr. 39, 1973–1983. doi: 10.1111/jbi.12000

[ref73] Wu D. Yu Q. Lu C. Hengsdijk H. (2006). Quantifying production potentials of winter wheat in the North China Plain. Eur. J. Agron. 24, 226–235. doi: 10.1016/j.eja.2005.06.001

[ref74] Yang Z. Zhou L. (2021). Is intestinal bacterial diversity enhanced by trans-species spread in the mixed-species flock of hooded crane (*Grus monacha*) and bean goose (*Anser fabalis*) wintering in the lower and middle Yangtze River floodplain? Animals 11:233. doi: 10.3390/ani11010233, PMID: 33477792 PMC7832407

[ref75] Zhang Y. Liu Y. Li J. Xing T. Jiang Y. Zhang L.. (2020). Dietary resistant starch modifies the composition and function of caecal microbiota of broilers. J. Sci. Food Agric. 100, 1274–1284. doi: 10.1002/jsfa.10139, PMID: 31721238

