# Supplementary material for: Comparative Analysis of the Gut Microbiota of Three Sympatric Terrestrial Wild Bird Species Overwintering in Farmland Habitats
Source: Front Microbiol. 2022 Jul 19;13:905668. doi: 10.3389/fmicb.2022.905668 (PMC9343720; doi:10.3389/fmicb.2022.905668)
Supplement: Supplementary file 1 [file Data_Sheet_1.docx]

Supplementary Materials

# Supplementary Figures


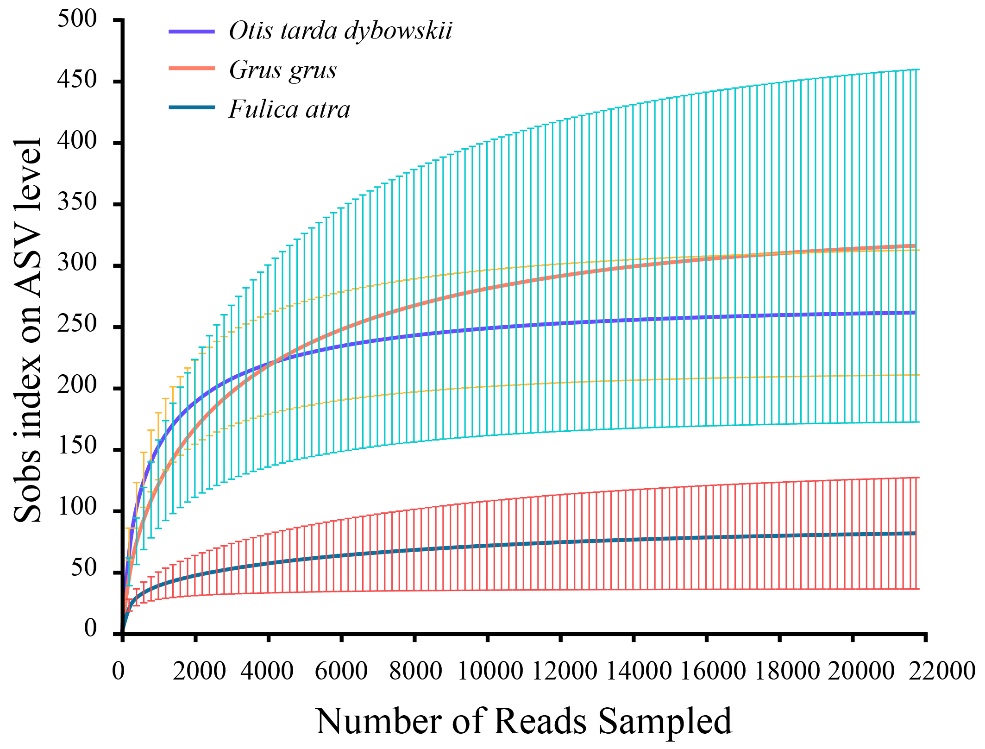


Figure S1. Average rarefaction curve representing variation in the Sobs index at increasing sequencing depth (21,753 reads only) of two groups. The error bars on the Sobs index correspond to the lower and upper bound 97% confidence intervals.


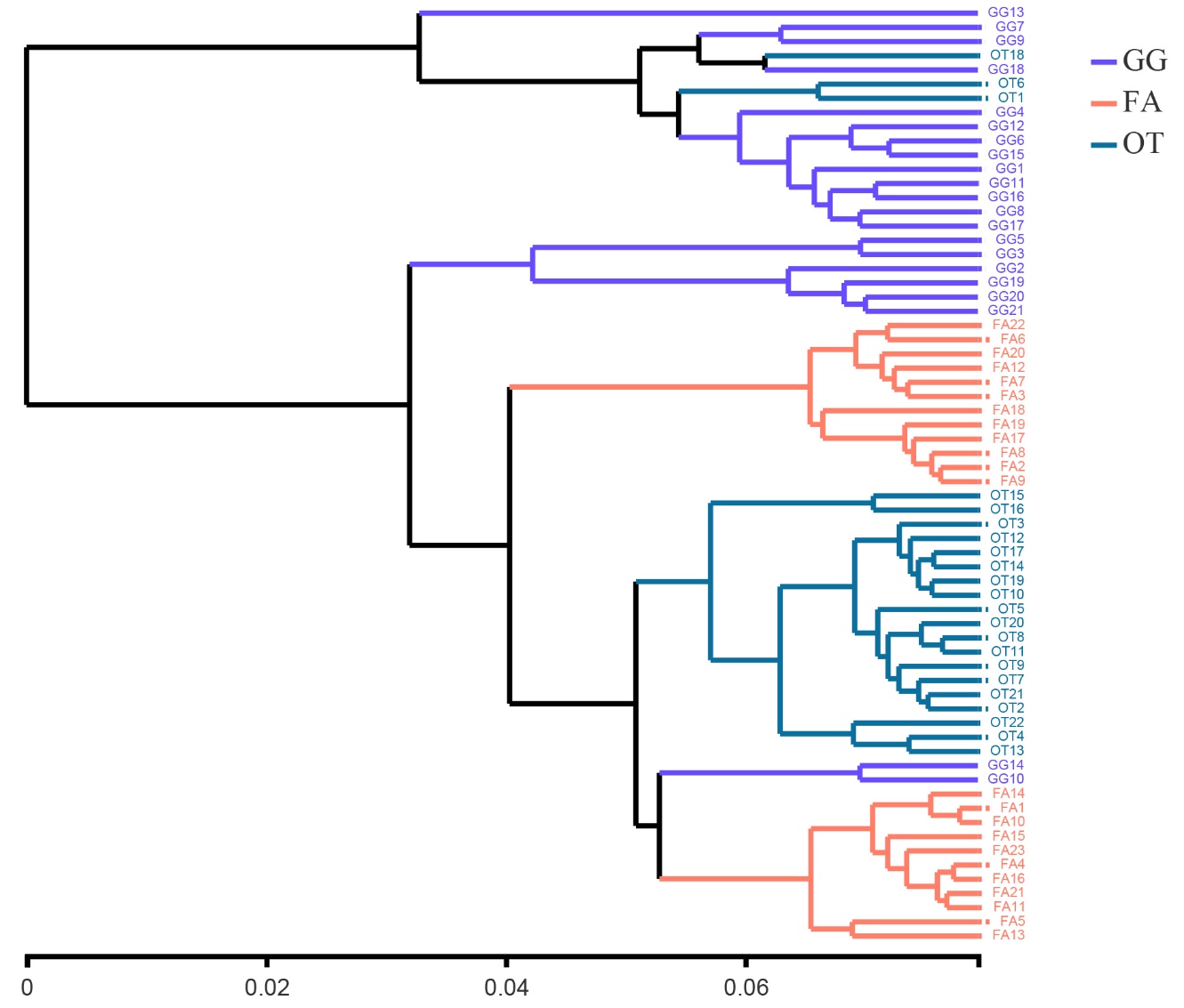


Figure S2. The hierarchical clustering tree shows the relative relationship of all samples based on the contained KEGG level Ⅱ pathways categories.

# Supplementary Tables

Table S1. The clean sequencing information of each sample.

| **Sample_info** | **ASVs_num** | **Seq_num** | **Sample_info** | **ASVs_num** | **Seq_num** |
| --- | --- | --- | --- | --- | --- |
| GG9 | 491 | 48479 | OT17 | 279 | 34029 |
| GG8 | 498 | 36706 | OT16 | 219 | 34868 |
| GG7 | 245 | 29308 | OT15 | 228 | 41470 |
| GG6 | 564 | 36512 | OT14 | 258 | 32946 |
| GG5 | 129 | 24875 | OT13 | 260 | 34382 |
| GG4 | 266 | 29741 | OT12 | 312 | 38972 |
| GG21 | 173 | 34248 | OT11 | 259 | 38123 |
| GG20 | 163 | 35712 | OT10 | 282 | 45820 |
| GG19 | 125 | 27778 | OT9 | 225 | 30487 |
| GG3 | 245 | 30051 | OT1 | 217 | 31042 |
| GG2 | 374 | 35279 | FA8 | 53 | 32359 |
| GG18 | 649 | 32634 | FA7 | 69 | 35016 |
| GG17 | 429 | 33234 | FA6 | 101 | 36716 |
| GG16 | 290 | 36648 | FA5 | 102 | 36038 |
| GG15 | 412 | 30932 | FA4 | 67 | 37673 |
| GG14 | 310 | 41137 | FA3 | 77 | 40058 |
| GG13 | 317 | 25721 | FA23 | 42 | 37922 |
| GG12 | 530 | 37366 | FA22 | 62 | 27348 |
| GG11 | 318 | 42967 | FA21 | 29 | 30713 |
| GG10 | 247 | 32769 | FA20 | 152 | 36464 |
| GG1 | 111 | 25721 | FA19 | 41 | 37167 |
| OT8 | 336 | 43824 | FA2 | 64 | 34645 |
| OT7 | 248 | 45352 | FA18 | 88 | 38292 |
| OT6 | 316 | 30446 | FA17 | 149 | 41753 |
| OT5 | 266 | 34262 | FA16 | 45 | 29071 |
| OT4 | 332 | 31230 | FA15 | 155 | 40508 |
| OT3 | 386 | 38742 | FA14 | 73 | 39534 |
| OT22 | 195 | 36219 | FA13 | 86 | 36056 |
| OT21 | 170 | 21753 | FA12 | 208 | 34301 |
| OT20 | 199 | 38652 | FA11 | 32 | 36127 |
| OT19 | 253 | 31719 | FA10 | 38 | 37406 |
| OT2 | 240 | 31930 | FA9 | 169 | 38806 |
| OT18 | 317 | 33337 | FA1 | 57 | 34304 |

Table S2. The Alpha diversity indexes of gut microbiota within each sample.

| **Sample** | **sobs** | **shannon** | **shannoneven** | **coverage** |
| --- | --- | --- | --- | --- |
| GG9 | 471 | 3.59137 | 0.583501 | 0.996966 |
| GG8 | 483 | 3.9147 | 0.633445 | 0.997242 |
| GG7 | 240 | 3.35133 | 0.611485 | 0.999586 |
| GG6 | 539 | 3.79958 | 0.604094 | 0.997518 |
| GG5 | 129 | 2.45809 | 0.505799 | 0.999954 |
| GG4 | 259 | 3.30904 | 0.595491 | 0.999448 |
| GG21 | 167 | 2.87858 | 0.562443 | 0.999218 |
| GG20 | 155 | 2.57963 | 0.511483 | 0.999218 |
| GG19 | 124 | 2.47921 | 0.514329 | 0.999724 |
| GG3 | 242 | 2.28347 | 0.416012 | 0.99954 |
| GG2 | 366 | 3.38248 | 0.573046 | 0.998759 |
| GG18 | 616 | 3.4357 | 0.534886 | 0.996414 |
| GG17 | 416 | 3.15607 | 0.523335 | 0.998023 |
| GG16 | 281 | 3.11713 | 0.552843 | 0.999081 |
| GG15 | 392 | 3.32776 | 0.557295 | 0.998253 |
| GG14 | 289 | 2.6614 | 0.469678 | 0.998437 |
| GG13 | 308 | 3.43336 | 0.59918 | 0.999908 |
| GG12 | 508 | 3.99238 | 0.640782 | 0.997885 |
| GG11 | 299 | 3.60718 | 0.632789 | 0.998483 |
| GG10 | 238 | 2.58819 | 0.472964 | 0.998989 |
| GG1 | 108 | 2.48546 | 0.530838 | 1 |
| OT8 | 331 | 4.66387 | 0.803821 | 0.999264 |
| OT7 | 241 | 3.86822 | 0.705263 | 0.999402 |
| OT6 | 314 | 3.98549 | 0.693202 | 0.999494 |
| OT5 | 266 | 3.67957 | 0.659009 | 0.999356 |
| OT4 | 329 | 4.34576 | 0.749778 | 0.999173 |
| OT3 | 381 | 4.72489 | 0.795061 | 0.999035 |
| OT22 | 194 | 2.98086 | 0.565857 | 0.999632 |
| OT21 | 170 | 3.33006 | 0.648401 | 1 |
| OT20 | 198 | 4.02414 | 0.760957 | 0.999862 |
| OT19 | 252 | 4.28259 | 0.774509 | 0.99977 |
| OT2 | 239 | 3.83949 | 0.70109 | 0.999908 |
| OT18 | 314 | 4.27091 | 0.742845 | 0.99931 |
| OT17 | 279 | 4.56954 | 0.811467 | 0.999724 |
| OT16 | 219 | 4.23077 | 0.785064 | 0.999862 |
| OT15 | 225 | 4.21314 | 0.777891 | 0.999724 |
| OT14 | 258 | 4.20574 | 0.757387 | 0.999678 |
| OT13 | 260 | 4.24783 | 0.763904 | 0.999218 |
| OT12 | 307 | 4.59705 | 0.802718 | 0.999724 |
| OT11 | 256 | 4.30843 | 0.776968 | 0.999678 |
| OT10 | 279 | 4.48146 | 0.795825 | 0.99954 |
| OT9 | 224 | 3.9285 | 0.725934 | 0.99977 |
| OT1 | 212 | 2.39168 | 0.446493 | 0.999218 |
| FA8 | 52 | 2.09169 | 0.529374 | 0.999816 |
| FA7 | 66 | 1.99745 | 0.476757 | 0.999632 |
| FA6 | 94 | 2.49003 | 0.548066 | 0.99954 |
| FA5 | 101 | 2.61445 | 0.566496 | 0.99954 |
| FA4 | 63 | 2.10215 | 0.507383 | 0.999724 |
| FA3 | 75 | 2.71053 | 0.627802 | 0.999402 |
| FA23 | 40 | 1.71967 | 0.466178 | 1 |
| FA22 | 60 | 2.25904 | 0.551747 | 0.999816 |
| FA21 | 28 | 1.62913 | 0.488903 | 0.999954 |
| FA20 | 147 | 2.80081 | 0.561236 | 0.999402 |
| FA19 | 40 | 1.89867 | 0.514702 | 0.999816 |
| FA2 | 63 | 2.16062 | 0.521494 | 0.999862 |
| FA18 | 82 | 2.57902 | 0.585246 | 0.99977 |
| FA17 | 141 | 2.70818 | 0.547244 | 0.999218 |
| FA16 | 44 | 1.9111 | 0.505023 | 0.999954 |
| FA15 | 150 | 2.47054 | 0.49306 | 0.998805 |
| FA14 | 70 | 2.24818 | 0.529171 | 0.999586 |
| FA13 | 85 | 2.58521 | 0.581907 | 0.999724 |
| FA12 | 197 | 2.7302 | 0.516769 | 0.998483 |
| FA11 | 30 | 1.9679 | 0.57859 | 1 |
| FA10 | 36 | 1.48525 | 0.414466 | 1 |
| FA9 | 155 | 2.65004 | 0.525444 | 0.998437 |
| FA1 | 55 | 2.00761 | 0.500984 | 0.999862 |

| **Genus** | **Common Coot** | | **Great Bustard** | | **Common Crane** | | **Reference** |
| --- | --- | --- | --- | --- | --- | --- | --- |
|  | Detection rate | Relative abundance (%) | Detection rate | Relative abundance (%) | Detection rate | Relative abundance (%) |  |
| *Campylobacter* | 23/23 | 3.2725 | 0/22 | 0 | 7/21 | 0.0050 | (Hubálek, 2004; Benskin et al., 2009) |
| *Escherichia-Shigella* | 11/23 | 0.0182 | 4/22 | 0.0276 | 3/21 | 0.0083 | (Laviad-Shitrit et al., 2019; Fu et al., 2020) |
| *Clostridium* | 4/23 | 0.0042 | 11/22 | 0.0890 | 8/21 | 0.0129 | (Fu et al., 2020; Yang and Zhou, 2021) |
| *Staphylococcus* | 0/23 | 0 | 0/22 | 0 | 10/21 | 0.0217 |  |
| *Vibrio* | 0/23 | 0 | 0/22 | 0 | 2/21 | 0.0007 | (Hubálek, 2004; Benskin et al., 2009) |
| *Macrococcus* | 0/23 | 0 | 0/22 | 0 | 10/21 | 0.0361 | (Yang and Zhou, 2021) |
| *Haemophilus* | 0/23 | 0 | 0/22 | 0 | 1/21 | 0.0004 | (Yang and Zhou, 2021) |
| *Yersinia* | 0/23 | 0 | 0/22 | 0 | 2/21 | 0.0018 | (Hubálek, 2004) |
| *Pasteurella* | 2/23 | 0.0018 | 0/22 | 0 | 0/21 | 0 | (Hubálek, 2004) |
| *Riemerella* | 0/23 | 0 | 0/22 | 0 | 1/21 | 0.0050 | (Hubálek, 2004) |
| *Erysipelothrix* | 0/23 | 0 | 0/22 | 0 | 1/21 | 0.0007 | (Hubálek, 2004) |
| *Mycobacterium* | 5/23 | 0.0052 | 0/22 | 0 | 21/21 | 0.2264 | (Hubálek, 2004) |
| *Helicobacter* | 4/23 | 0.1919 | 1/22 | 0.0017 | 1/21 | 0.0011 | (Ryu et al., 2014) |

Table S3. Potentially pathogenic genera were detected in the host samples.

References:

Benskin, C. McW. H., Wilson, K., Jones, K., and Hartley, I. R. (2009). Bacterial pathogens in wild birds: a review of the frequency and effects of infection. *Biological Reviews* 84, 349–373. doi: 10.1111/j.1469-185X.2008.00076.x.

Fu, R., Xiang, X., Dong, Y., Cheng, L., and Zhou, L. (2020). Comparing the intestinal bacterial communies of sympatric wintering Hooded Crane (*Grus monacha*) and Domestic Goose (*Anser anser domesticus*). *Avian Res* 11, 1–9. doi: 10.1186/s40657-020-00195-9.

Hubálek, Z. (2004). AN ANNOTATED CHECKLIST OF PATHOGENIC MICROORGANISMS ASSOCIATED WITH MIGRATORY BIRDS. *Journal of Wildlife Diseases* 40, 639–659. doi: 10.7589/0090-3558-40.4.639.

Laviad-Shitrit, S., Izhaki, I., Lalzar, M., and Halpern, M. (2019). Comparative Analysis of Intestine Microbiota of Four Wild Waterbird Species. *Front Microbiol* 10, 1911. doi: 10.3389/fmicb.2019.01911.

Ryu, H., Grond, K., Verheijen, B., Elk, M., Buehler, D. M., and Domingo, J. W. S. (2014). Intestinal Microbiota and Species Diversity of *Campylobacter* and *Helicobacter* spp. in Migrating Shorebirds in Delaware Bay. *Appl Environ Microb* 80, 10. doi:10.1128/AEM.03793-13.

Yang, Z., and Zhou, L. (2021). Is Intestinal Bacterial Diversity Enhanced by Trans-Species Spread in the Mixed-Species Flock of Hooded Crane (Grus monacha) and Bean Goose (Anser fabalis) Wintering in the Lower and Middle Yangtze River Floodplain? *Animals* 11, 233. doi: 10.3390/ani11010233.

Table S4. The statistical test of the difference in Alpha diversity indexes between groups.

| **estimators** | **OT-mean** | **OT-sd** | **FA-mean** | **FA-sd** | **GG-mean** | **GG-sd** | **statistic** | **pvalue** | **qvalue** | **Pvalue(FA-GG)** | **Pvalue(OT-FA)** | **Pvalue(OT-GG)** |
| --- | --- | --- | --- | --- | --- | --- | --- | --- | --- | --- | --- | --- |
| sobs | 261.3 | 52.05 | 81.48 | 46.41 | 315.7 | 147.1 | 41.2104 | 1.13E-09 | 3.67E-09 | < 0.001 | < 0.001 | >= 0.1 |
| shannon | 4.053 | 0.5612 | 2.253 | 0.3886 | 3.135 | 0.5246 | 43.8415 | 3.02E-10 | 2.72E-09 | < 0.001 | < 0.001 | < 0.001 |
| shannoneven | 0.7292 | 0.08748 | 0.5277 | 0.0461 | 0.5536 | 0.05967 | 35.3507 | 2.11E-08 | 3.16E-08 | >= 0.1 | < 0.001 | < 0.001 |

Table S5. ANOSIM analysis among three wild birds.

| **Species** | **R statistic** | **Significance level** |
| --- | --- | --- |
| Great Bustard vs Common Crane vs Common Coot | 0.9982 | 0.001 |
| Great Bustard vs Common Crane | 0.9996 | 0.001 |
| Great Bustard vs Common Coot | 1 | 0.001 |
| Common Crane vs Common Coot | 0.9949 | 0.001 |
